# *Staphylococcus aureus*-specific IgA antibody in milk suppresses the multiplication of *S. aureus* in infected bovine udder

**DOI:** 10.1186/s12917-019-2025-3

**Published:** 2019-08-09

**Authors:** Yuya Nagasawa, Yoshio Kiku, Kazue Sugawara, Aya Hirose, Chiaki Kai, Nana Kitano, Toshihiko Takahashi, Tomonori Nochi, Hisashi Aso, Shin-ichi Sawada, Kazunari Akiyoshi, Tomohito Hayashi

**Affiliations:** 1 Dairy Hygiene Unit, Division of Pathology and Pathophysiology, Hokkaido Research Station, National Institute of Animal Health, National Agriculture and Food Research Organization, 4 Hitsujigaoka, Toyohira, Sapporo, Hokkaido 062-0045 Japan; 2 Graduate school of Dairy Science, Rakuno Gakuen University, 582, Bunkyodai-Midorimachi, Ebetsu, Hokkaido 069-8501 Japan; 3 Cellular Biology Laboratory, Graduate School of Agricultural Science, Tohoku University, Sendai, Miyagi 980-0845 Japan; 4 Department of Polymer Chemistry, Graduate School of Engineering, Kyoto University, Katsura, Nishikyo-ku, Kyoto, 615-8510 Japan

**Keywords:** *Staphylococcus aureus*, IgA, Milk, Bovine mastitis

## Abstract

**Background:**

Bovine mastitis caused by *Staphylococcus aureus* (*S. aureus*) is extremely difficult to control and new methods for its prevention and management are required. Nasal vaccines may prevent initial bovine mastitis infection caused by *S. aureus*. However, limited information is available regarding induction of mucosal immune response through nasal immunization with antigen and its suppression of *S. aureus* multiplication during bovine mastitis. This study sought to investigate whether induction of immunoglobulin A (IgA) in milk by nasal immunization could suppress multiplication of *S. aureus* in the bovine udder.

**Results:**

Nasal immunization with formalin-killed *S. aureus* conjugated with a cationic cholesteryl-group-bearing pullulan-nanogel was performed. Anti-*S. aureus*-specific IgA antibodies were significantly more abundant in the milk of immunized cows than in non-immunized animals (*P* < 0.05). *S. aureus* counts in the quarter were negative in both non-immunized and nasal-immunized cows 1 week after mock infusion. In *S. aureus*-infused quarters, *S. aureus* multiplication was significantly suppressed in immunized compared with non-immunized cows (*P* < 0.05). Furthermore, a significant negative correlation was found between *S. aureus*-specific IgA antibodies and *S. aureus* counts in infused quarters of both non-immunized and nasal-immunized cows (r = − 0.811, *P* < 0.01).

**Conclusion:**

In conclusion, the present study demonstrates that *S. aureus*-specific IgA antibodies in milk successfully suppressed the multiplication of *S. aureus* in infected bovine udders. Although the exact mechanism explaining such suppressive effect remains to be elucidated, nasal vaccines that can induce humoral immunity may help prevent initial infection with *S. aureus* and the onset of bovine mastitis.

**Electronic supplementary material:**

The online version of this article (10.1186/s12917-019-2025-3) contains supplementary material, which is available to authorized users.

## Background

Bovine mastitis refers to inflammation of the mammary gland and can be caused by several bacterial species [1, 2]. It is a complex disease and a major source of economic loss for the dairy industry [1, 3]. *Staphylococcus aureus* is the most common etiologic agent of chronic, contagious, and intractable bovine mastitis [4, 5]. Because *S. aureus*-derived bovine mastitis is extremely difficult to control, new methods aimed at its prevention and containment are required. Vaccination is an effective strategy to prevent inflammation, including bovine mastitis. Even though vaccines are used globally to protect cows from mastitis caused by *S. aureus* [6, 7], they are not sufficiently effective at preventing onset of the disease and minimizing its global economic fallout [8, 9]. Therefore, further research on improved effectiveness of the vaccine is required.

Systemic immunization ensures that adequate amounts of antigen reach peripheral lymphoid tissues and protect against infectious agents, however it is largely ineffective in providing immunity to mucosal surfaces [10]. To this end, mucosal vaccines have been proposed recently as effective control methods [11]. They induce a mucosal immune response by preventing the entry of pathogens into the body through the mucosal surface. Mucosal membranes can secrete immunoglobulin A (IgA) antibodies to block or inactivate pathogens [12]. Therefore, mucosal immune responses can function as an important first line of defence on the mucosal surface. Mucosal vaccines have been designed based on the premise that antigen uptake at inductive sites results in the dissemination of IgA-committed plasma cells to remote mucosal tissues and glands [13, 14]. In humans, protective mucosal immune responses are induced most substantially by mucosal immunization through the oral or nasal routes [15]. In the case of ruminants, the nasal cavity is one of the most promising administration sites because the oral route has high digestive enzymatic activity [16, 17] and may dissolve vaccine antigens. Thus, nasal vaccines may help prevent initial infection with *S. aureus* and avoid bovine mastitis. However, limited information is available regarding induction of mucosal immune responses through nasal immunization with antigen and its ability to suppress the multiplication of *S. aureus* in udders with bovine mastitis.

The objective of this study was to investigate whether induction of IgA in milk by nasal mucosa immunization could suppress multiplication of *S. aureus* in the bovine udder. Considering that mucosa-administered antigens are generally less immunogenic and may induce tolerance, potent mucosal adjuvants, vectors, or other special delivery systems are required for successful mucosal vaccination [18]. In this context, the use of toxin-based adjuvants is undesirable as it carries the risk that the toxin may reach the central nervous system [19, 20]. Moreover, following the failure of single antigen vaccine approaches against *S. aureus*, most efforts are now focused towards multiple antigen strategies [21]. To overcome these concerns, we used formalin-killed *S. aureus* (FKSA) supplied via an intranasal vaccine-delivery system with a nanometre-sized hydrogel consisting of cationic type of cholesteryl group bearing pullulan (cCHP) [22]. cCHP nanogels have been reported to interact with proteins and cell membranes through hydrophobic and electrostatic interactions [22]. The same study also suggested that cCHP nanogels formed complexes with FKSA, which could interact with the nasal mucosa. Therefore, to confirm that cCHP nanogel/inactivated *S. aureus* complexes could serve as a delivery system for mucosal immunization, we compared the uptake of inactivated *S. aureus* alone and cCHP nanogel/inactivated *S. aureus* complexes in Waldeyer’s ring. cCHP nanogel facilitated early antigen uptake by enhancing delivery and adherence of the vaccine antigen to the nasal epithelium. In a previous study, high-magnification images showed that cCHP/botulinum type A neurotoxin was internalized into the nasal epithelium within 1 h following nasal administration and that it gradually detached from cCHP nanogel in nasal epithelial cells [22]. In addition, ex vivo uptake of microparticles by bovine pharyngeal tonsils revealed macrophages with intracellular beads in the deep regions of the epithelial layer 1 h after administration [23]. Based on the above evidence, we focused on events occurring 1 h after nasal administration. Experimental challenge studies with *S. aureus* have shown an effect of vaccination on the amount of bacterial shedding after the challenge [24]. Therefore, after nasal immunization of cows with inactivated *S. aureus*, we used experimental models of mastitis through mammary infection with *S. aureus* and investigated *S. aureus-*specific IgA antibody and *S. aureus* counts in milk. This study sets the basis for the future development of an efficient nasal vaccine against bovine mastitis.

## Results

### cCHP nanogel/*S. aureus*-conjugated FITC (SA-FITC) is taken up significantly more than SA-FITC alone in tissues from Waldeyer’s ring

**Fig. 1:**
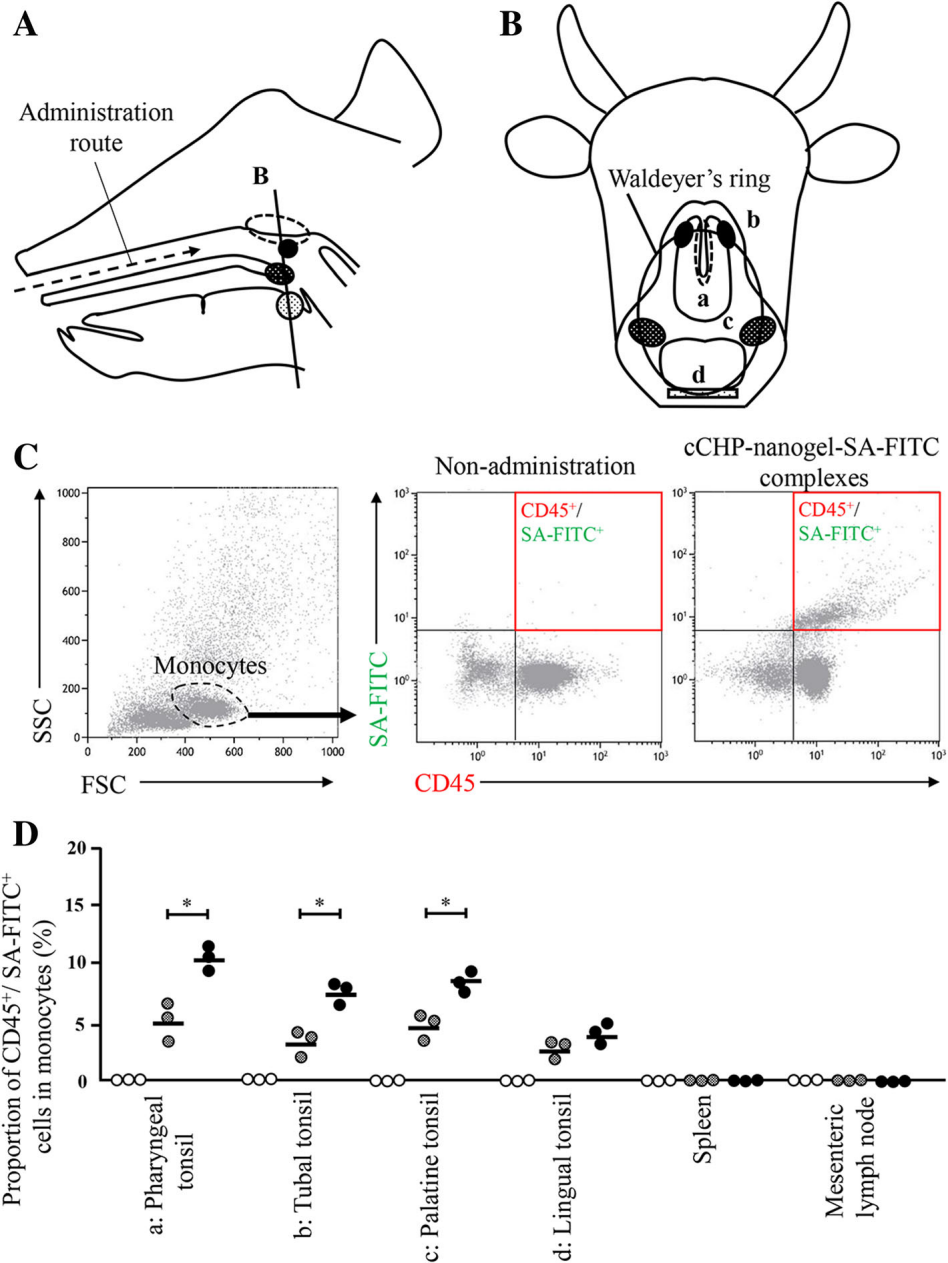
Distribution of CD45^+^ cells in calves lymphoid pharyngeal tissues (Waldeyer’s ring), 60 min after intranasal administration of *S. aureus*-conjugated FITC. After nasal administration with *S. aureus*-conjugated FITC (SA-FITC) or cCHP nanogel-SA-FITC complexes, FITC^+^/CD45^+^ mononuclear cells of Waldeyer’s ring, spleen, and mesenteric lymph node in calves were evaluated by flow cytometry. **a** Transversal section (plane indicated as B) of Waldeyer’s ring and route of administration with SA-FITC or cCHP nanogel/SA-FITC complexes. **b** Location of the pharyngeal tonsils (**a**), paired tubal tonsils (**b**), paired palatine tonsils (**c**), and lingual tonsil (**d**). **c** Gating scheme for the identification of CD45^+^ leukocytes based on forward and side light scatter characteristics of tissue monocytes. **d** Dot plot showing the percentage of FITC^+^/CD45^+^ mononuclear cells in pharyngeal tonsils (**a**), paired tubal tonsils (**b**), paired palatine tonsils (**c**), lingual tonsil (**d**), spleen, and mesenteric lymph node. Black circles correspond to nasal immunization with cCHP nanogel/SA-FITC complexes, grey circles to nasal immunization with SA-FITC, and white circles to non-immunization controls. *Significant difference between the mean of each group (*P* < 0.05)

In calves given intranasal SA-FITC alone or cCHP nanogel/SA-FITC complexes, FITC^+^/ CD45^+^ cells were detected in the pharyngeal tonsil, tubal tonsil, palatine tonsil, and lingual tonsil, but not in the spleen and mesenteric lymph node. In particular, FITC^+^/CD45^+^ cells in the pharyngeal tonsil, tubal tonsil, and palatine tonsil were significantly higher following administration of cCHP nanogel/SA-FITC complexes than SA-FITC alone (mean % was 10.6 vs. 5.0 for pharyngeal tonsil; 7.2 vs. 3.2 for tubal tonsil; and 7.8 vs. 4.9 for palatine tonsil; *P* < 0.05) (Fig. 1d).

### Anti-*S. aureus*-specific IgA and immunoglobulin G (IgG) antibodies in milk increases following nasal immunization with FKSA

**Fig. 2:**
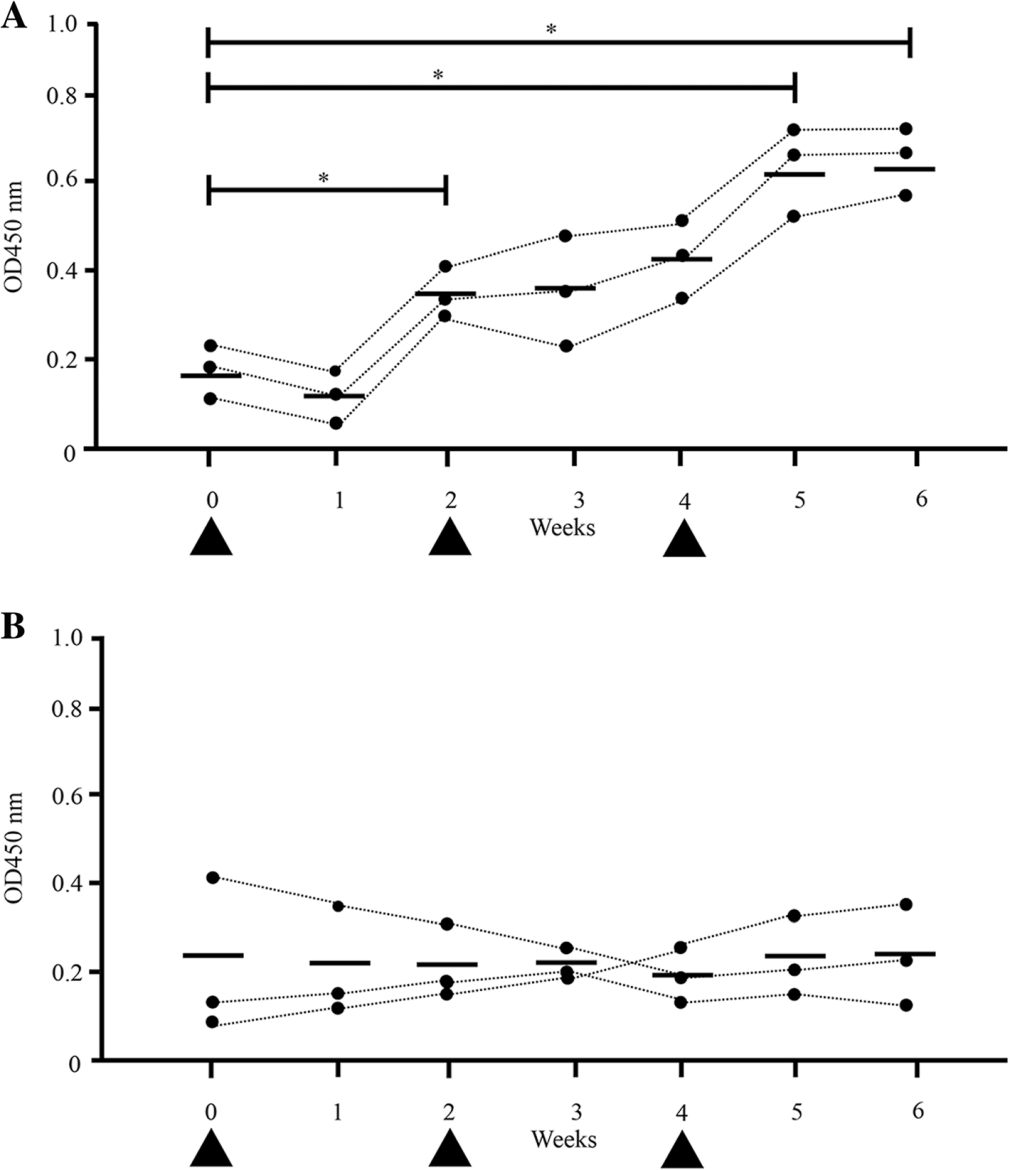
Anti-*S. aureus*-specific IgA and IgG antibodies in milk from cows immunized with FKSA coupled with cCHP nanogel, administered intranasally in 2 weeks intervals in three subsequent doses (OD values). **a** OD values of anti-*S. aureus*-specific IgA antibodies in composite milk samples (three cows, *n* = 3) from cows that underwent repeated nasal immunization with FKSA three times with a two-week interval. **b** Anti-*S. aureus*-specific IgG antibodies. Each black circle corresponds to a sample and the bar represents the mean. Nasal immunizations are indicated by arrowheads. *Significant difference between the mean of each group on day 0 and from one to 6 weeks after immunization (*P* < 0.05)

To confirm increased presence of *S. aureus*-specific IgA antibodies in milk, three cows were repeatedly immunized with FKSA. The optical density (OD) values of anti-*S. aureus*-specific IgA antibodies in composite milk (three cows, *n* = 3) were significantly higher two, five, and 6 weeks after immunization than before immunization (mean ODs were 0.38, 0.61, 0.62, and 0.18, respectively; *P* < 0.05) (Fig. 2a); whereas OD values of anti-*S. aureus*-specific IgG antibodies in composite milk (three cows, n = 3) were not significantly changed (Fig. 2b).

### Anti-*S. aureus*-specific IgA antibodies in milk is higher in immunized than in non-immunized cows

**Fig. 3:**
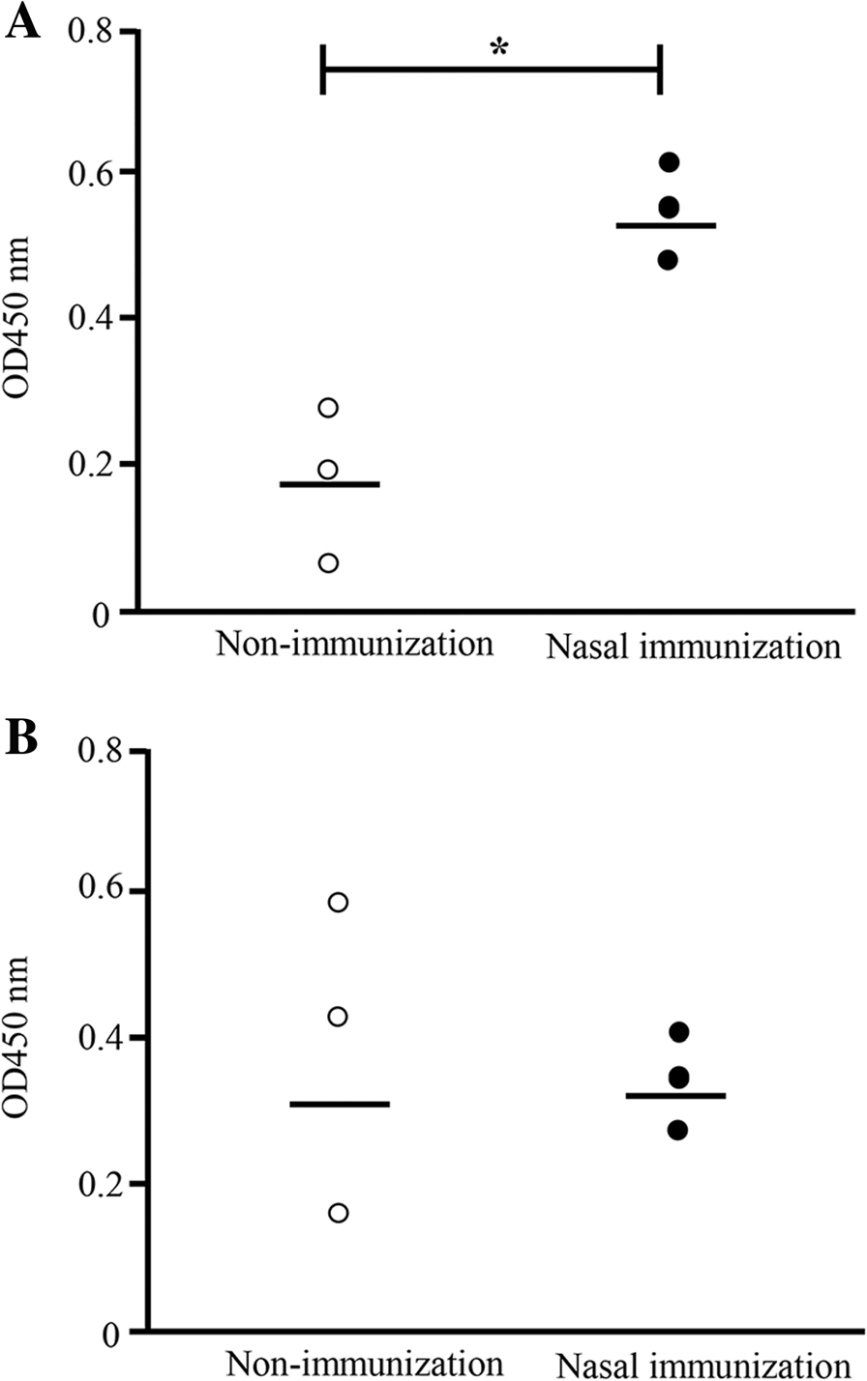
Anti-*S. aureus*-specific IgA and IgG antibodies in milk from immunized and non-immunized cows (OD values). After 4 weeks following nasal immunization (10 weeks after day 0 of pre-immunization = day 0 of pre-infusion), cows were tested for anti-*S. aureus*-specific IgA and IgG antibodies in milk of immunized and non-immunized cows (**a**) OD values of anti-*S. aureus*-specific IgA antibodies in milk after nasal immunization with FKSA or no immunization. **b** Anti-*S. aureus*-specific IgG antibodies. Each data point represents the OD value for one composite milk sample (three cows, n = 3), and the bar represents the mean. Black circles correspond to nasal immunization samples, white circles to non-immunization controls. *Significant difference between the mean of each group (*P* < 0.05)

On pre-infusion day (day 0), after nasal immunization with FKSA, OD values of anti-*S. aureus*-specific IgA antibodies were significantly higher in composite milk from immunized cows than in milk from non-immunized controls (mean ODs were 0.50 vs. 0.18, respectively; *P* < 0.05) (Fig. 3a); whereas OD values of anti-*S. aureus*-specific IgG antibodies in milk did not differ significantly between non-immunized and nasal-immunized cows (Fig. 3b).

### Anti-*S. aureus*-specific IgA antibodies in milk increases following infusion with *S. aureus* BM1006

**Fig. 4:**
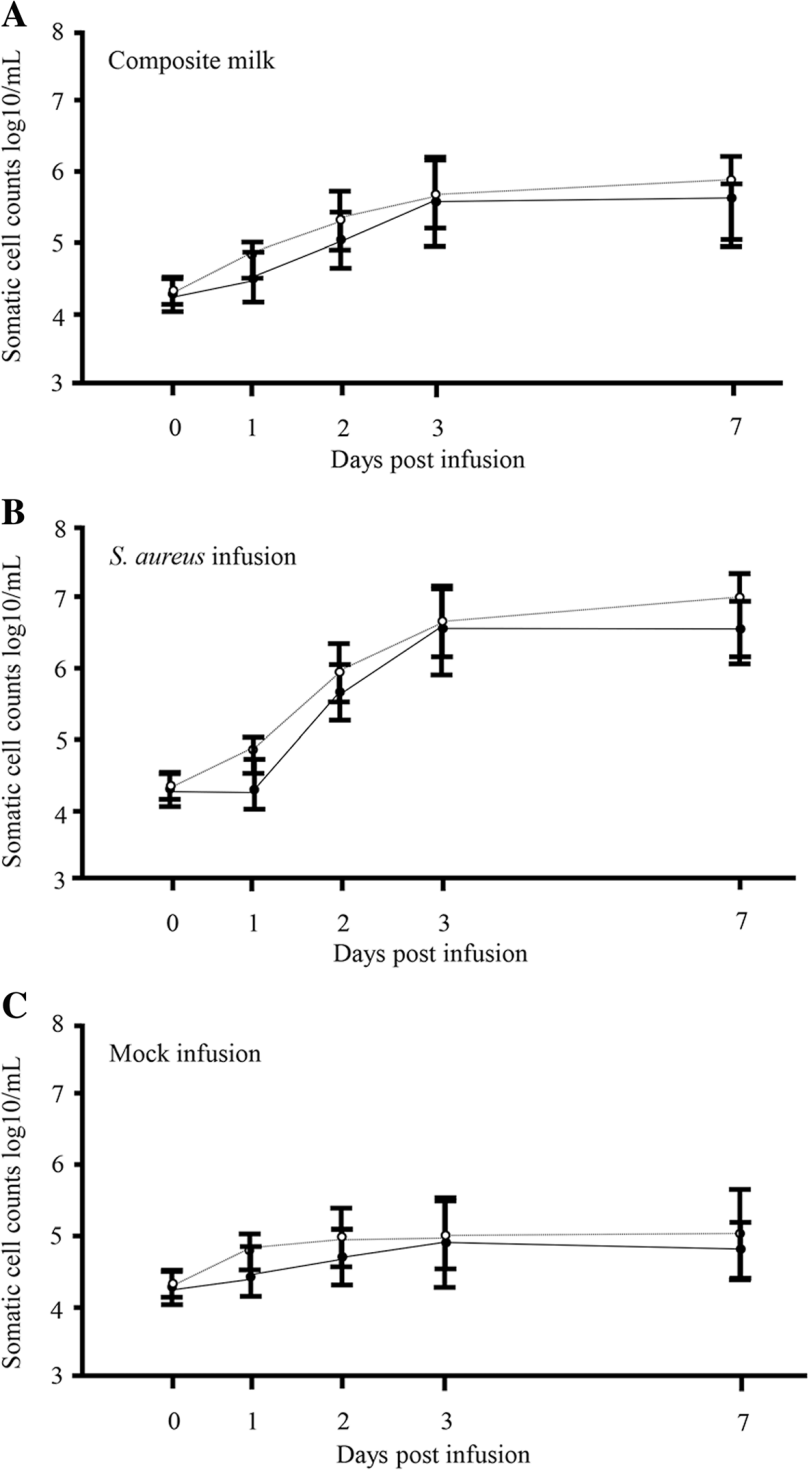
Somatic cell count in milk following quarters’ infusion with *S. aureus* BM1006. Somatic cell counts in composite milk and quarter milk following nasal immunization with FKSA or no immunization in the early stage of lactation, and mock-infusion with PBS or infusion with *S. aureus* BM1006 in the gland cistern. **a** Composite milk (three cows, n = 3). **b** Two quarters infused with *S. aureus* BM1006 in the gland cistern (six quarters, *n* = 6). **c** Two quarters mock-infused with PBS (six quarters, n = 6). Mean cell counts (± standard error) are shown. Black circles correspond to nasal immunization samples, white circles to non-immunization controls

**Fig. 5:**
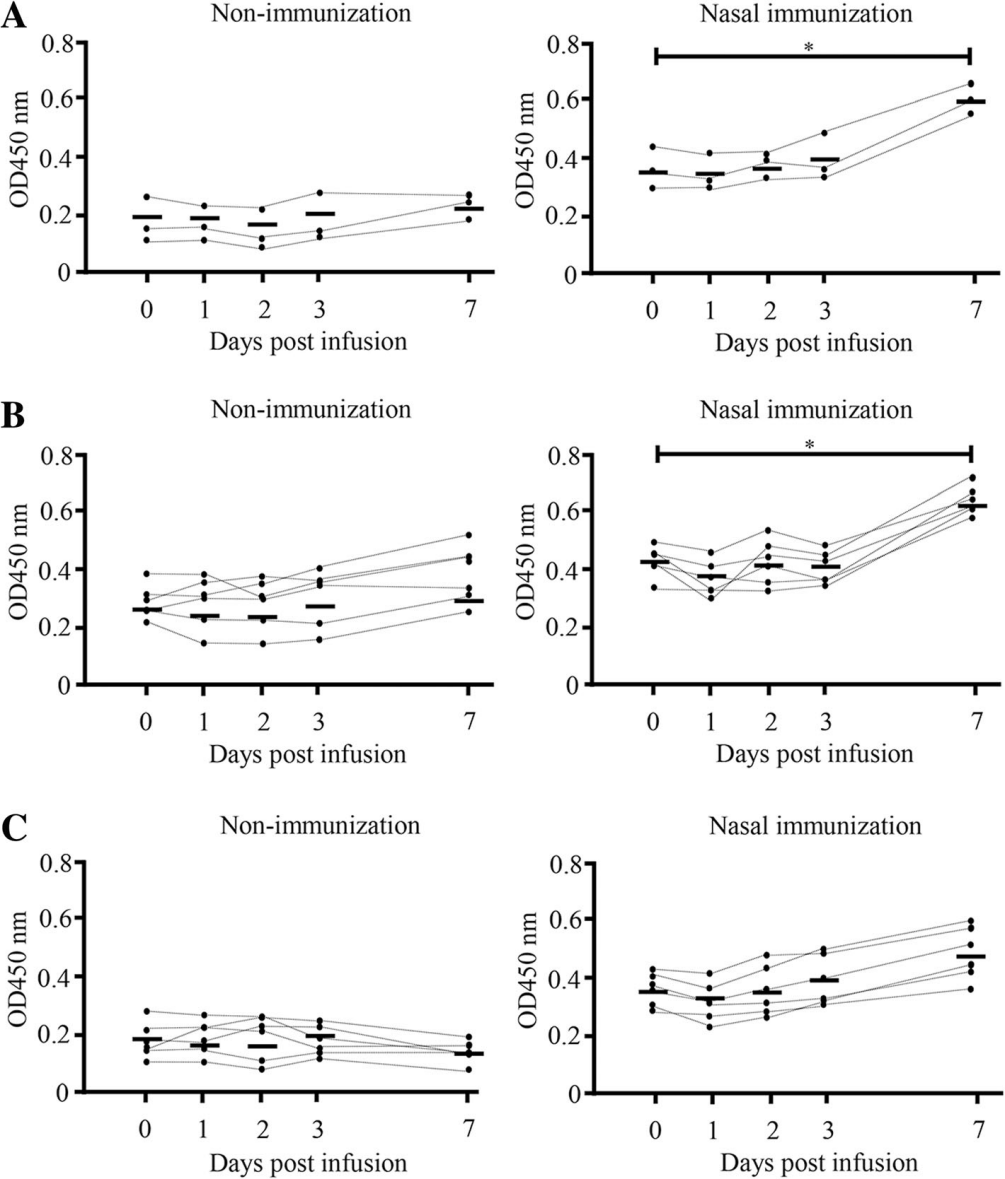
Anti-*S. aureus*-specific IgA antibodies in milk (OD values) following quarters’ infusion with *S. aureus* BM1006. OD values of *S. aureus*-specific IgA antibodies in milk following nasal immunization with FKSA or no immunization in the early stage of lactation. **a** Composite milk (three cows, n = 3). **b** Two quarters infused with *S. aureus* BM1006 in the gland cistern (six quarters, n = 6). **c** Two quarters mock-infused with PBS (six quarters, n = 6). Each data point represents the OD value for one sample, and the bar represents the mean. Black circles correspond to nasal immunization samples, white circles to non-immunization controls. *Significant difference between the mean on day 0 and day 7 after infusion in the same group (*P* < 0.05)

**Fig. 6:**
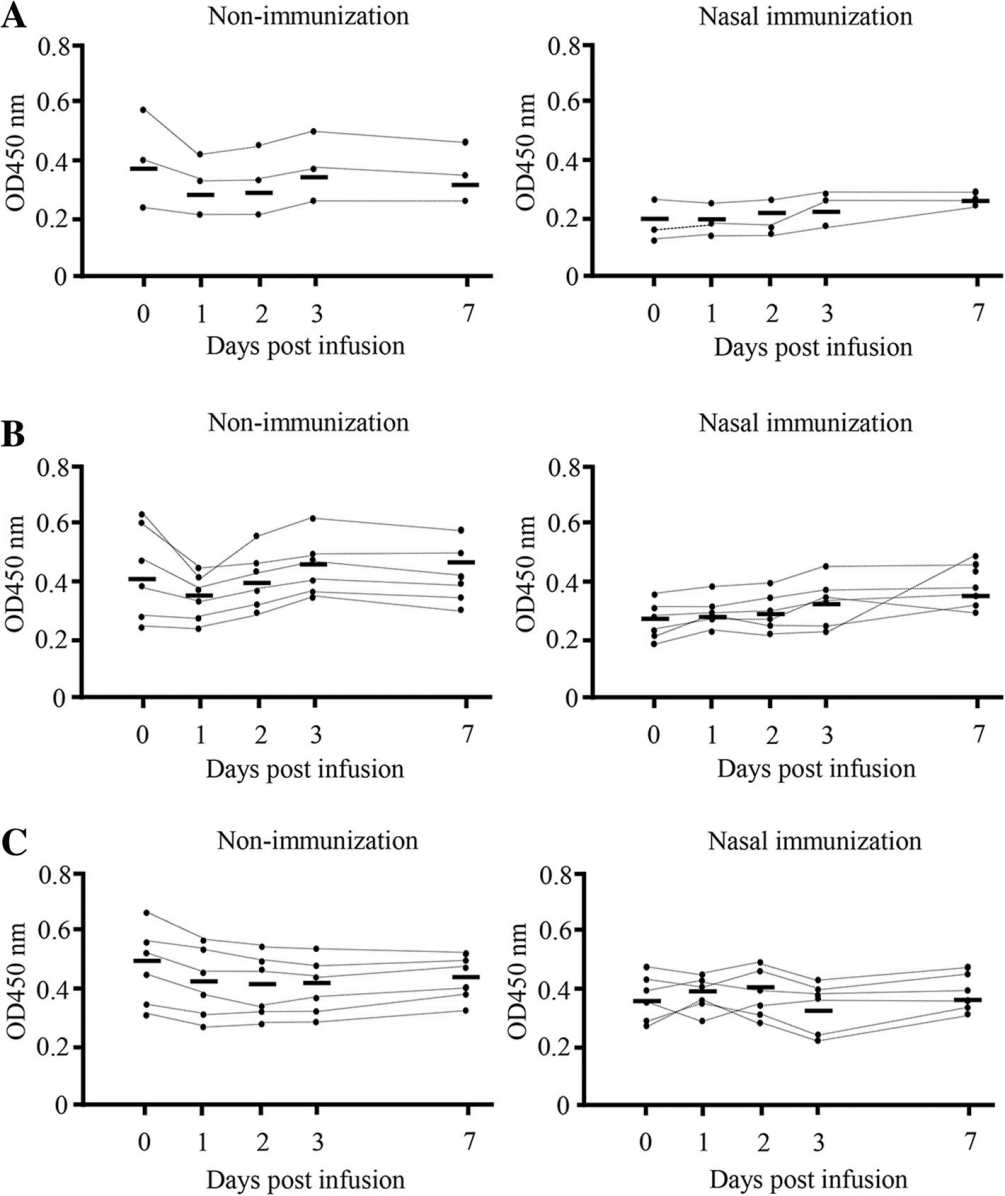
Anti-*S. aureus*-specific IgG antibodies in milk (OD values) following quarters infusion with *S. aureus* BM1006. OD values of *S. aureus*-specific IgG antibodies in milk following nasal immunization with FKSA or no immunization in the early stage of lactation. **a** Composite milk (three cows, n = 3). **b** Two quarters infused with *S. aureus* BM1006 in the gland cistern (six quarters, n = 6). **c** Two quarters mock-infused with PBS (six quarters, n = 6). Each data point represents the OD value for one sample, and the bar represents the mean. Black circles correspond to nasal immunization samples, white circles to non-immunization controls

After *S. aureus* infusion, somatic cell count (SCC) increased dramatically and peaked at 3 days for composite milk and *S. aureus*-infused quarter; whereas no significant change was detected following mock infusion. No significant differences in SCC could be detected between day 0 and day 7 in non-immunized and nasal-immunized animals (Fig. 4). Three to seven days after *S. aureus* infusion, all the cows exhibited localized signs of clinical mastitis, including clots in the milk, swelling of the udders, and a loss of milk yield. One of the non-immunized cows presented also systemic signs, including elevated rectal temperature and loss of appetite. We then investigated the OD value of *S. aureus*-specific IgA and IgG antibodies and *S. aureus* counts in milk from these cows (Fig. 5). In non-infused quarters of non-immunized and nasal-immunized animals (three cows, six quarters, *n* = 6, respectively), no significant differences in *S. aureus*-specific IgA antibodies could be detected between day 0 and 7 days post-mock infusion (Fig. 5c). In contrast, in composite milk (three cows, *n* = 3) and infused quarters (three cows, six quarters, *n* = 6) from immunized cows, the OD value increased significantly 7 days after infusion compared with day 0 (mean of composite milk = 0.64 vs. 0.41, mean of *S. aureus*-infused quarter = 0.72 vs. 0.42, respectively; *P* < 0.05) (Fig. 5a and b). The values were not significantly different in non-immunized cows (three cows, six quarters, *n* = 6) (Fig. 5a, b, and c). No significant differences in *S. aureus*-specific IgG antibodies could be detected between day 0 and day 7 in composite milk, post-*S. aureus-* and mock-infused quarters of non-immunized and nasal-immunized animals (Fig. 6a, b, and c).

### *S. aureus* counts in milk from pre-immunized and non-immunized cows decrease 1 week after intramammary *S. aureus* infusion

**Fig. 7:**
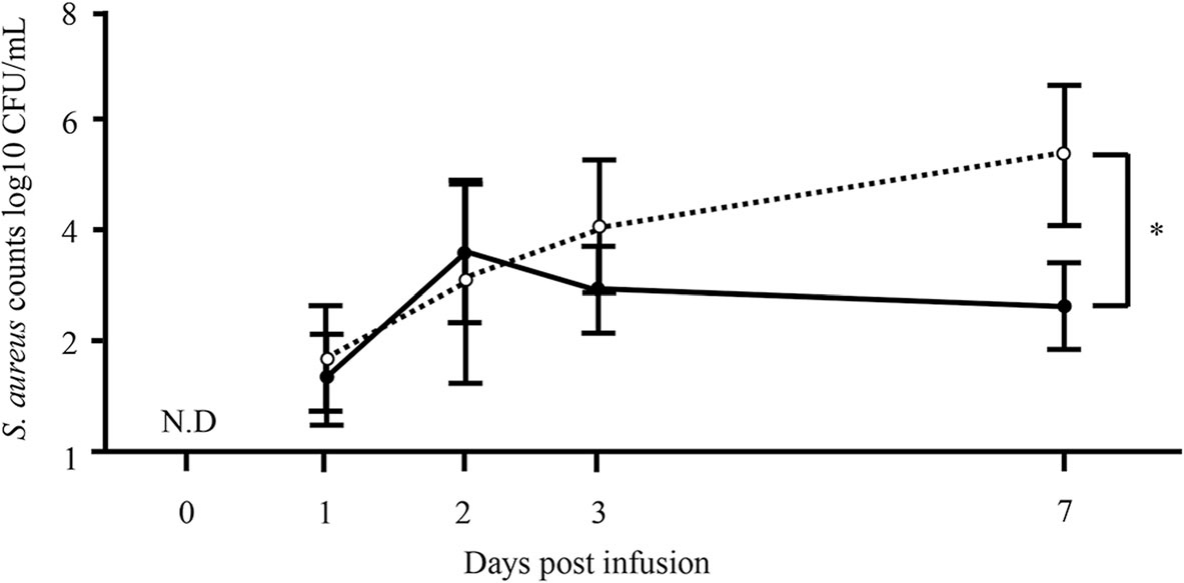
*S. aureus* counts in milk 1 week after experimental intramammary infection of immunized and non-immunized cows. *S. aureus* counts in milk were determined in immunized and non-immunized cows from pre-infusion on day 0 to day 1, 2, 3, and 7 after *S. aureus* infusion into the quarter (three cows, 6 quarters, n = 6). Mean (± standard error) *S. aureus* counts are shown. Black circles correspond to nasal immunization samples, white circles to non-immunization controls. *Significant difference between the mean of each group (*P* < 0.05). N.D, not detected

At pre-infusion and n days 1, 2, 3, and 7 after mock infusion, *S. aureus* counts could not be detected in either non-immunized or nasal-immunized cows (three cows, six quarters, n = 6, respectively, data not shown). However, multiplying *S. aureus* could be detected in infused quarters in both non-immunized and nasal-immunized cows 7 days after infusion (three cows, six quarters, n = 6, respectively). In particular, *S. aureus* was significantly suppressed in immunized compared with non-immunized cows (mean of *S. aureus* counts expressed as log10 colony-forming units (CFU)/mL was 3.7 vs. 5.2, respectively; *P* < 0.05) (Fig. 7).

### *S. aureus* counts correlate negatively with anti-*S. aureus*-specific IgA antibodies in milk

**Fig. 8:**
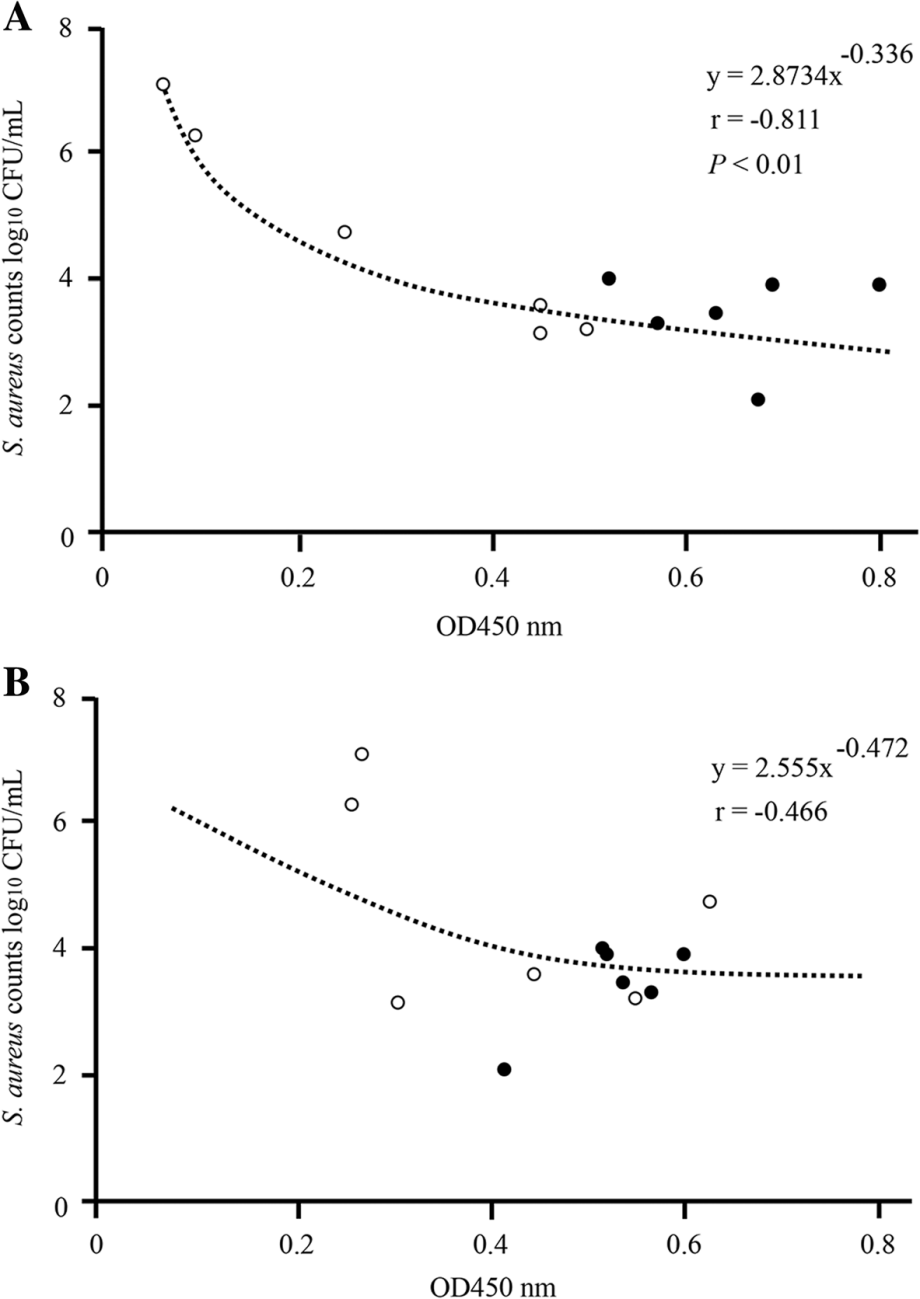
*S. aureus* counts and anti-*S. aureus*-specific IgA or IgG antibodies in milk a week after experimental intramammary infection of immunized and non-immunized cows. **a** Linear regression analysis showing a statistical correlation between *S. aureus* counts and OD value of *S. aureus*-specific IgA antibodies in infused quarters of non-immunized and nasal-immunized cows (six cows, 12 quarters, *n* = 12). **b** *S. aureus*-specific IgG antibodies. Black circles correspond to nasal immunization samples, white circles to non-immunization controls (three cows, six quarters, n = 6). *P* < 0.01 was considered highly statistically significant

To evaluate the effect of IgA antibodies on the suppressed multiplication of *S. aureus* in milk, a correlation between the two values 1 week after *S. aureus* infusion was analysed by the Pearson correlation coefficient. A significant negative correlation was found between *S. aureus*-specific IgA antibodies and *S. aureus* counts in infused quarters of both non-immunized and nasal-immunized cows (six cows, 12 quarters, *n* = 12, r = − 0.811, *P* < 0.01) (Fig. 8a), but no statistical correlation was found for *S. aureus*-specific IgG antibodies (six cows, 12 quarters, n = 12, r = − 0.466, *P* < 0.01) (Fig. 8b).

## Discussion

In this study, we investigated whether *S. aureus*-specific IgA antibodies in milk induced by intranasal *S. aureus* immunization suppressed the multiplication of intramammary *S. aureus*. Initially, we investigated the ability of cCHP nanogel to complex with FKSA. Fluorescence intensity was higher at 0.05 mg/mL than at 0.02 mg/mL cCHP-Rh. cCHP nanogel consists of the cationic molecular ethylenediamine modified cholesteryl pullulan, and previous studies have demonstrated that cationization of nanogel increases the binding constant between nanogels and anionic proteins such as bovine serum albumin [25]. This is due to both hydrophobic and electrostatic interactions between cationic nanogels and bovine serum albumin. The surface of *S. aureus*, like that of most bacteria, has a negative charge [26] and, therefore, is thought to form a complex with the positively charged cationic nanogel.

Antigen proteins complexed with the cCHP nanogel possess a positive charge, possibly promoting adhesion to the negatively charged surface of the nasal mucosa. Kopatz et al. demonstrated that electrostatic interaction on anionic heparan sulphate proteoglycans played an important role in the uptake of cationic PEI/DNA complexes by the mucosal surface [27]. In addition, cCHP/botulinum type A neurotoxin bound electrostatically to the negatively charged nasopharynx-associated lymphoid tissue (NALT) was found to be responsible for the initiation of antigen-specific immune responses [22]. Therefore, cCHP nanogel complexed with inactivated *S. aureus* could adhere continuously to the nasal mucosa. So far, there have been no reports on the interaction of *S. aureus* complexed with cCHP nanogel to the mucosa, including the lymphoid tissues of Waldeyer’s ring. In particular, pharyngeal tonsils are considered to be the most important tissue for the immune response due to the presence of microfold cells that take up foreign antigens [23]. Hence, we focused on Waldeyer’s ring as an immune response inductive site. Interestingly, flow cytometry detected FITC^+^/CD45^+^ cells only in the tissues of Waldeyer’s ring but not in physically distant tissues, such as the spleen and mesenteric lymph nodes (Fig. 1d). Because CD45 is a marker expressed on all leukocytes, which plays an important role in signal transduction in T cells, intranasally administered bacteria were likely taken up by local leukocytes. Our result suggests that Waldeyer’s ring is the first site of recognition of inhaled pathogens. Moreover, FITC^+^/CD45^+^ cells in the pharyngeal tonsil, tubal tonsil, and palatine tonsil were significantly more abundant when administered as cCHP nanogel/SA-FITC than as SA-FITC alone (Fig. 1d). These results also suggested that the cationization of *S. aureus* by complexing with cCHP nanogel enhanced antigen uptake by leukocytes of Waldeyer’s ring. Therefore, cCHP nanogel can represent a suitable delivery system for mucosal immunization, including with nasal immunization antigens. Time-dependent kinetics of CD45^+^/FITC^+^ cells will have to be determined to further characterize the properties of cCHP nanogel.

Next, cows received nasal immunization with FKSA three times with a two-week interval to induce the production of *S. aureus*-specific IgA antibodies. Results demonstrate that the OD value of *S. aureus*-specific IgA antibody in milk was significantly higher in nasal-immunized cows than in pre-immunized control animals (Figs. 2 and 3). Immune responses generated in NALT provide long-term protection against respiratory disease [28, 29]. In the case of cows, inactivated vaccines and modified live virus vaccines are administered via the intranasal route to prevent BHV-1 infections [30–32]. In addition, the nasal cavity is one of the most promising administration sites because the oral route has high digestive enzymatic activity and may dissolve vaccine antigens. Interestingly, a previous study reported that cows, which were immunized via the mucosa (including intranasally) with inactivated *S. aureus* strains, presented *S. aureus*-specific IgA antibodies in the milk [33], confirming that nasal immunization with inactivated *S. aureus* could actually induce *S. aureus*-specific IgA antibodies in the mammary gland. In contrast, mucosal immunization generally induces mucosal-specific IgA antibodies and serum-specific IgG antibodies; however, the OD value of anti-*S. aureus*-specific IgG antibodies in composite milk did not differ significantly in this study (Fig. 2). This phenomenon may be related to differences in adjuvant. Nanoparticulate carriers such as cCHP nanogel provide adjuvant activity by enhancing antigen delivery or activating innate immune responses. Strength and mechanisms of immunostimulation induced by nanocarrier vaccines depend on various factors, such as chemical composition, particle size, homogeneity, and electrical charge [34–36]. Misstear et al. [37] demonstrated that T helper 1- and T helper 17-biased responses elicited by targeted nanoparticles using intranasal vaccination with microfold cell-targeted ovalbumin-loaded nanoparticles provided protection against *S. aureus*, without the induction of any detectable antigen-specific serum IgG. At the same time, Boerhout et al. suggested that intranasal mucosal immunization by spray resulted in a marginal response only, indicating that this route is less suitable for generating humoral protection against *S. aureus* [38]. This discrepancy with respect to our results may be due to the different administration method. Bovine mucosa-associated lymphoid tissues in the upper respiratory tract include NALT and the lymphoid tissues of Waldeyer’s ring, which itself encompasses the pharyngeal, palatine, tubal, and lingual tonsils. Even though NALT is located near the nasal cavity, there have been only a few studies on it in cows [39]. Therefore, to make sure that FKSA were delivered to Waldeyer’s ring located in the deep part of the nasal cavity, cattle were treated intranasally with a ~ 15-cm feeding needle for rats, rather than a spray commonly used for nasal administration. Future studies should focus on the type of adjuvant to be used for delivery and its ability to stimulate various pathways.

Next, after nasal immunization of inactivated *S. aureus* to cows, we used experimental models of mastitis through mammary infection with *S. aureus* and investigated *S. aureus-*specific IgA and IgG antibodies and *S. aureus* counts in milk. In composite and *S. aureus*-infused quarter milk from immunized cows, IgA antibodies increased significantly on day 7 after infusion compared with day 0 (Fig. 5a and b), but no significant differences in *S. aureus*-specific IgG antibodies could be detected between days 0 and 7 (Fig. 6a and b). In addition, in non-infused quarters, there was no significant difference in *S. aureus*-specific IgA and IgG antibodies between day 0 and day 7, irrespective of immunization (Figs. 5c and 6c). Leitner et al. [40] indicated that IgA antibodies were the major Ig isotype in most *S. aureus*-infected quarters with subclinical chronic mastitis but not in non-infected quarters. These results suggest that production of the IgA isotype is localized to individual quarters and nasal immunization can provide local immunity against *S. aureus* infection. Similarly, average *S. aureus* counts in infused quarters were significantly lower in immunized cows than in non-immunized animals (Fig. 7). Unlike IgGs, the IgA isotype is not among the immunoglobulin classes in milk that promote phagocytosis [41, 42]. However, IgA antibodies may control the severity of the initial infection via immune exclusion, thus preventing bacteria from adhering to the epithelial cells on the mucosal surface [12]. In fact, our present study confirms a significant negative correlation between OD value of *S. aureus*-specific IgA antibodies and *S. aureus* counts (Fig. 8a), but a statistical correlation was not found for *S. aureus*-specific IgG antibodies (Fig. 8b). Accordingly, induction of *S. aureus*-specific IgA antibodies positively affects prevention and control of *S. aureus* infection in the quarter. In addition, our previous study showed a significant correlation between *S. aureus* counts and the proportion of mammary epithelial cells in milk, thus suggesting that *S. aureus* counts in milk reflected exfoliation of mammary epithelial cells related to mammary damage [43]. These reports suggested that reduction of *S. aureus* counts through induction of IgA antibodies could protect cells from initial infection by *S. aureus,* including in cases of mammary damage. Present results suggest that induction of *S. aureus*-specific IgA antibodies by nasal immunization is beneficial for the clearance of *S. aureus* from the udder.

Based on our results, *S. aureus* in milk was still detected in immunized cows, in spite of a significant negative correlation between *S. aureus*-specific IgA antibodies and *S. aureus* counts (Fig. 8a). This result indicates that although nasal immunization can suppress the multiplication of *S. aureus* and limit new infections, complete control was not achieved. Compared to other bacterial pathogens, *S. aureus* presents several challenges. Targeting specific antigens has been suggested as the most efficient method to develop an effective vaccine. However, other studies suggest that *S. aureus* milk isolates have a large polymorphism and regional patterns, stressing the importance of developing vaccines based on antigens common to different isolates. Accordingly, no optimal vaccine antigen against *S. aureus* could be achieved, making it difficult to control the multiplication of this bacterium. At the same time, it should be noted that previous studies focused mainly on IgG antibodies and, particularly in cows, research on induction of IgA antibodies is still limited. Until recently, anti-*S. aureus* vaccine approaches have focused on induction of neutralizing/opsonizing antibodies by IgG, but there is increasing evidence that cellular immunity may be equally or more important for protective immunity [44]. Indeed, mucosal sites are responsible for the generation of antigen-specific T helper 2-dependent IgA responses and T helper 1- and cytotoxic T lymphocyte-dependent immune responses, which function as the first line of defence on mucosal surfaces. In many cases of mucosal vaccines, the main protective effector function elicited by immunization is stimulation of a secretory local IgA antibody response and an associated mucosal immunologic memory. However, in other instances, although still less extensively studied, there is evidence of an important role also for the cellular arm of the immune response that includes mucosal CD8^+^ cytotoxic T lymphocytes, CD4^+^ T helper cells, as well as natural killer cells [45]. Remarkably, Misstear et al. [37] demonstrated that a targeted nasal vaccine promoted clearance of an acute *S. aureus* systemic infection, but also that a purely cellular response was sufficient for this protection in mice. It is of strategic importance that future research focuses on the search for effective vaccine antigens against bovine mastitis, which can be applied via the mucosal route. Moreover, it will be essential to assess functional responses through induction of both humoral and cellular immunity, including T cell-mediated responses.

## Conclusion

In conclusion, the present study demonstrates that induction of *S. aureus*-specific IgA antibodies in milk by nasal immunization suppressed multiplication of *S. aureus* in the udder. Although future research will need to clarify the exact mechanism by which IgA antibodies suppress bacterial multiplication, our findings support the ongoing effort to develop a mucosal vaccine against bovine *S. aureus*-induced mastitis and indicate that stimulation of an anti-*S. aureus* humoral immune response in milk might contribute to prevention and control of the disease.

## Methods

### Cattle

Nine 6 to 10-week-old male calves and six 16 to 28-month-old Holstein primiparous heifers during early lactation were obtained from dairy farms in Hokkaido, Japan. None of the animals used in the present study had any previous history of clinical or subclinical mastitis. Four weeks after parturition, three cows were used for intranasal immunization with FKSA. During the experiment, all cows were kept in a biosafety level 2 animal facility at the Hokkaido Research Station, National Institute of Animal Health (Sapporo, Hokkaido, Japan). The following criteria, which correspond to the Japanese diagnostic standard for bovine mastitis, were used for quarter milk samples: negative for *S. aureus* infection and SCC < 3 × 10^5^ cells/mL.

### Preparation of cCHP nanogel/inactivated *S. aureus* complexes

*S. aureus* strain BM1006 (sequence type 352, clonal complex 97), which causes bovine mastitis [46], was used to prepare FKSA as described in our previous study [47]. Washed *S. aureus* BM1006 was suspended in phosphate-buffered saline (PBS) containing 0.5% formaldehyde, incubated with shaking (50 rpm) at room temperature for inactivation, and then washed three times with PBS. We confirmed the absence of viable cells after 1 h following inactivation (Additional file 1). The inactivation step was then performed overnight to ensure complete inactivation.

cCHP, or cCHP-rhodamine (cCHP-Rh) were synthesized as described previously [22, 25, 48]. cCHP or cCHP-Rh were suspended in PBS. The suspension was sonicated for 15 min with a probe sonicator (BRANSON, Danbury, CT, USA) and centrifuged at 20,000×*g* for 30 min. The obtained supernatant was then filtered through a 0.22-μm filter (Millipore, Billerica, MA, USA). To confirm whether cCHP nanogel reliably complexed with FKSA, we incubated 5 × 10^9^ FKSA cells/mL with cCHP-Rh nanogel (final concentration 0.02, 0.05, or 0.2 mg/mL) for 30 min at room temperature, and then observed the samples under a confocal laser microscope (LSM780, Carl Zeiss, Göttingen, Germany). Images revealed that FKSA was not sufficiently complexed by the cCHP-Rh nanogel at 0.02 mg/mL; however, complexation was successful at 0.05 mg/mL, as indicated by substantially stronger fluorescence intensity of the 0.05 mg/mL sample. Conversely, there was no major difference in fluorescence intensity between this and the 0.2 mg/mL sample (Additional file 2). To ensure that the cCHP nanogel complexed with FKSA, 5 × 10^10^ FKSA cells were incubated for 30 min at room temperature with cCHP nanogel at a final concentration of 1 mg/mL. The resulting cCHP nanogel/inactivated *S. aureus* complexes were suspended in 2 mL PBS and used for intranasal immunization.

### Nasal administration of cCHP nanogel/inactivated *S. aureus*-conjugated FITC to calves

SA-FITC was purchased from Thermo Fisher Scientific (S2851, Waltham, MA, USA). As above, 3 × 10^9^ SA-FITC cells were incubated for 30 min with cCHP nanogel at a final concentration of 1 mg/mL. The resulting cCHP nanogel/SA-FITC complexes, as well as SA-FITC alone (3 × 10^9^ cells/2 mL, respectively), were administered with a ~ 15-cm flexible feeding needle to each of three male calves into one nostril (Fig. 1a). One hour after nasal administration, calves were injected xylazine (Selactar 2% injection solution, Bayer Yakuhin, Tokyo, Japan) as a sedative, general anaesthesia was induced by intravenous sodium pentobarbital (Somnopentyl, Kyoritsu Seiyaku, Tokyo, Japan), and calves were sacrificed by exsanguination from the carotid artery. To confirm that the cells had taken up SA-FITC, tissues surrounding Waldeyer’s ring (pharyngeal tonsil, tubal tonsil, palatine tonsil, and lingual tonsil) (Fig. 1b), the spleen, and the mesenteric lymph node were collected after sacrifice and analysed by flow cytometry. Three calves not treated with either SA-FITC or cCHP nanogel/SA-FITC were sacrificed and tissues from the same sites were used as negative controls.

### Flow cytometry analysis

Flow cytometry was performed on lymphocytes isolated from the above-mentioned tissues. All tissues were finely cut, transferred into PBS containing 1 mg/mL collagenase (034-22363, Wako, Osaka, Japan), and incubated at 37 °C for 10 min in a shaking water bath. The digested tissues were washed with PBS and filtered through a 40-μm stainless steel mesh to separate the cells from the excess tissue debris. To detect leukocytes, 1 × 10^6^ leukocytes were incubated with rabbit anti-CD45 antibody (1:200; Abcam, Cambridge, MA, USA) for 60 min, rinsed, and then incubated with Alexa Fluor 647 donkey anti-rabbit IgG antibody (1:200; Molecular Probes, Leiden, The Netherlands) for 1 h. Finally, the cell suspensions were fixed in 500 μL of 1% formalin in PBS. The number and percentage of FITC^+^/CD45^+^ mononuclear cells in tissue was analysed on a Gallios™ flow cytometer (Beckman Coulter Inc., Fullerton, CA, USA) based on forward and side light scattering properties (Fig. 1c). A total of 10^4^ events per sample were collected, and positive cells were expressed as a proportion of mononuclear cells. Rabbit IgG isotype controls (Abcam) were used to detect non-specific staining and establish the criteria for positive cell populations (data not shown).

### Nasal immunization with cCHP nanogel/inactivated *S. aureus* complexes

Three Holstein cows were intranasally immunized three times to one nostril with FKSA-complexed cCHP nanogel (5 × 10^10^ cells/2 mL) with a two-week interval between each dose. Following nasal immunization, the cows were clinically evaluated to determine their wellbeing in terms of local reactions of the quarter (quarter uniformity, milk abnormalities, appetite, and milk yield) and systemic reactions (rectal temperature and appetite). No significant SCC in milk (Additional file 3), systemic or local reactions were observed (data not shown). After 4 weeks following nasal immunization, cows were tested for experimental intramammary infusion with *S. aureus*.

### Experimental intramammary infusion with *S. aureus*

Six cows (three non-intranasally or three intranasally cows) in the early stage of lactation were intramammary infused with *S. aureus* BM1006. Before infusion, we conducted a bacteriological examination of milk samples, which were found to be completely negative for *S. aureus*. Intramammary infusion with *S. aureus* was performed as described in our previous study [43]. Briefly, *S. aureus* was diluted to ~ 20 CFUs in a total of 5 mL. Two quarters from each cow were selected after evening milking. Teats were allowed to air-dry, and a *S. aureus* suspension was infused into the gland cistern of two quarters, whereas the remaining two quarters were mock-challenged with the same volume of PBS. Clinical signs such as rectal temperature, udder uniformity, milk abnormalities, appetite, and milk yield for each cow were recorded in the morning.

### Milk sample collection and *S. aureus* counts

Milk samples were collected from day 0 of pre-immunization every week for up to 6 weeks, and from day 0 of pre-infusion until day 1, 2, 3, and 7 after infusion with *S. aureus.* Samples were collected from each individual quarter and composite milk. Composite milk samples refer to milk aliquots collected from all four quarters into a single sample vial [49, 50]. A composite milk sample (40 mL) was created by mixing milk from each of the four quarters into a sterile test tube. A 1-mL aliquot of each milk sample was spread on a Petrifilm Staph Express Count plate (3 M, Minneapolis, MN, USA) and incubated at 37 °C for 24 h. Then, CFUs were counted. When colony colours other than red-violet were present, the plates were applied to a Petrifilm Staph Express Disk and incubated for 3 h. The colonies that formed pink zones were counted as *S. aureus*, and the results were expressed as CFU/mL. The SCC of the milk was measured using a DeLaval cell counter DCC (DeLaval, Tumba, Sweden) as described by Kawai et al. [51].

### The enzyme-linked immunosorbent assay (ELISA) for the detection of specific IgA and IgG antibodies against *S. aureus*

To detect specific IgA and IgG antibodies against *S. aureus* in individual quarter and composite milk, a microtiter plate was directly coated with FKSA as capture antigen. FKSA in PBS was dried in an oven at 5 × 10^6^ cells/well in 96-well ELISA plates (C96 Maxisorpcert, Nunc-Immuno Plate, Thermo Fisher Scientific) overnight at 37 °C. After incubation, wells were washed with Tris-buffered saline-Tween-20 (TBST) and then incubated with 100 μL of milk (diluted 1:100 in PBS) for 90 min at room temperature. After five TBST washes, wells were incubated with horseradish peroxidase-conjugated sheep anti-bovine IgA antibody (diluted 1:30000, Bethyl Laboratories, Inc., Montgomery, TX, USA) or horseradish peroxidase-conjugated sheep anti-bovine IgG antibody (diluted 1:30000, Bethyl Laboratories, Inc.) for 2 h at room temperature. The freshly prepared substrate was added and OD was measured at 450 nm using the 3′,3′,5,5′-tetramethylbenzidine microwell peroxidase substrate system (KPL, Gaithersburg, MD, USA). All samples were analysed in duplicate and mean values were calculated. Anti-*S. aureus*-IgA or IgG antibodies were calculated by subtracting OD values for the buffer controls (OD450 nm = ~ 0.15), which were included in duplicate in all ELISAs, from specific sample OD values. To standardize and compare results between plates, positive control milk samples (from bovine mastitis caused by *S. aureus*, OD450 nm = ~ 1.5 for specific IgA antibody, OD450 nm = ~ 1.0 for specific IgG) were included in duplicate in all ELISAs. OD values were normalized against those of positive controls.

### Statistical analysis

Differences among groups of FITC^+^/CD45^+^ mononuclear cells, SCC, *S. aureus* counts, and the OD value of *S. aureus*-specific IgA and IgG were evaluated using Student’s *t*-test. Associations between *S. aureus* counts and antibody OD values were analysed by applying linear mixed models with SAS software (SAS Institute Japan Ltd., Tokyo, Japan). Pearson correlation coefficient and nonlinear regression analysis were used to assess the relationship between *S. aureus* counts and OD value of anti-*S. aureus*-specific IgA or IgG antibodies. *P* < 0.05 and *P* < 0.01 were considered significant and highly significant, respectively.

## Additional files

Additional file 1: Survival curve of *Staphylococcus aureus* BM1006 strain inactivated with 0.5% formaldehyde. *S. aureus* BM1006 (5 × 10^10^ CFUs) was incubated with 0.5% formaldehyde for 0, 15, 30, 180, and 1080 min, and then the number of colonies was counted using a Petrifilm Staph Express Count plate. Data are presented as logarithmic bacterial reduction in log CFU/mL. Black circles represent the mean of five independent experiments, error bars indicate the standard deviation. (TIF 91 kb)

Additional file 2: Confocal fluorescence images of inactivated *Staphylococcus aureus* BM1006 strain incubated with cCHP nanogel. Formalin-killed *S. aureus* BM1006 samples (FKSA, 5 × 10^9^ cells/mL) were incubated for 30 min at room temperature with 0.02, 0.05, or 0.2 mg/mL cCHP-Rh nanogel, and then observed using a confocal laser microscope. Bright-field, cCHP-Rh, and merge images are shown. Scale bars, 5 μm. Arrows indicate FKSA not complexed by the cCHP-Rh nanogel. (TIF 428 kb)

Additional file 3: Somatic cell count in milk following nasal immunization with FKSA. After nasal immunization with FKSA or no immunization, somatic cell count (SCC) in composite and quarter milk was analysed. Each data point represents the SCC for composite milk sample (three cows, *n* = 3) and quarter milk samples (three cows, 12 quarters, *n* = 12); the bar represents the mean. Black circles correspond to nasal immunization samples. (TIF 82 kb)

## Data Availability

The dataset supporting the conclusions of this article is included within the article.

## Abbreviations

cCHP: cationic type of cholesteryl-group-bearing pullulan
CFU: Colony-forming unit
ELISA: Enzyme-linked immunosorbent assay
FKSA: Formalin-killed *Staphylococcus aureus*
IgA: Immunoglobulin A
IgG: Immunoglobulin G
NALT: Nasopharynx-associated lymphoid tissue
OD: Optical density
PBS: Phosphate-buffered saline
SCC: Somatic cell count
TBST: Tris-buffered saline-Tween-20
