# Supplementary figures and images for: Staphylococcus aureus-specific IgA antibody in milk suppresses the multiplication of S. aureus in infected bovine udder
Source: BMC Vet Res. 2019 Aug 9;15:286. doi: 10.1186/s12917-019-2025-3 (PMC6688226; doi:10.1186/s12917-019-2025-3)

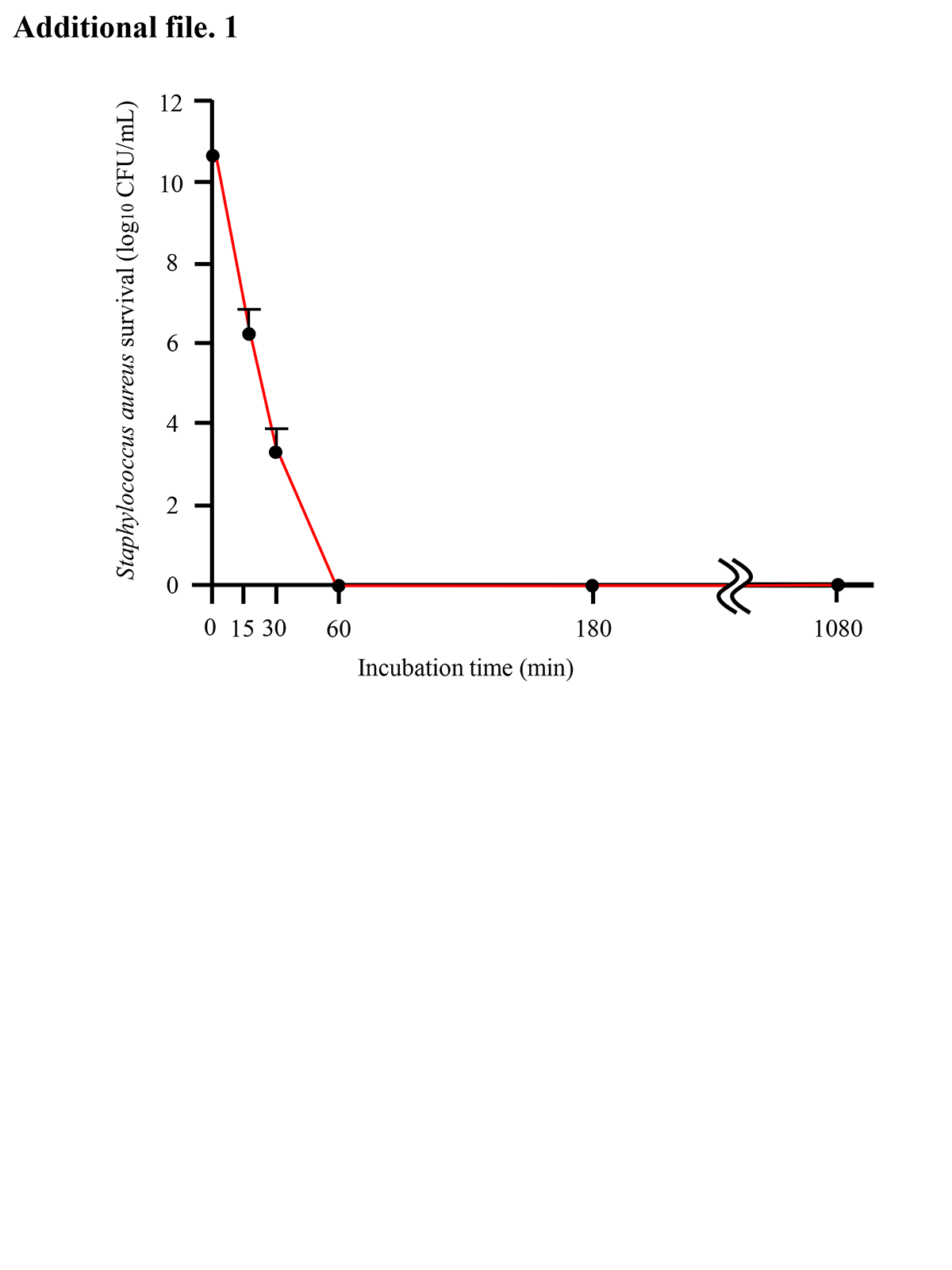

Supplement: Supplementary file 1 — Survival curve of Staphylococcus aureus BM1006 strain inactivated with 0.5% formaldehyde. S. aureus BM1006 (5 × 10^10^ CFUs) was incubated with 0.5% formaldehyde for 0, 15, 30, 180, and 1080 min, and then the number of colonies was counted using a Petrifilm Staph Express Count plate. Data are presented as logarithmic bacterial reduction in log CFU/mL. Black circles represent the mean of five independent experiments, error bars indicate the standard deviation. (TIF 91 kb) [file 12917_2019_2025_MOESM1_ESM.tif]

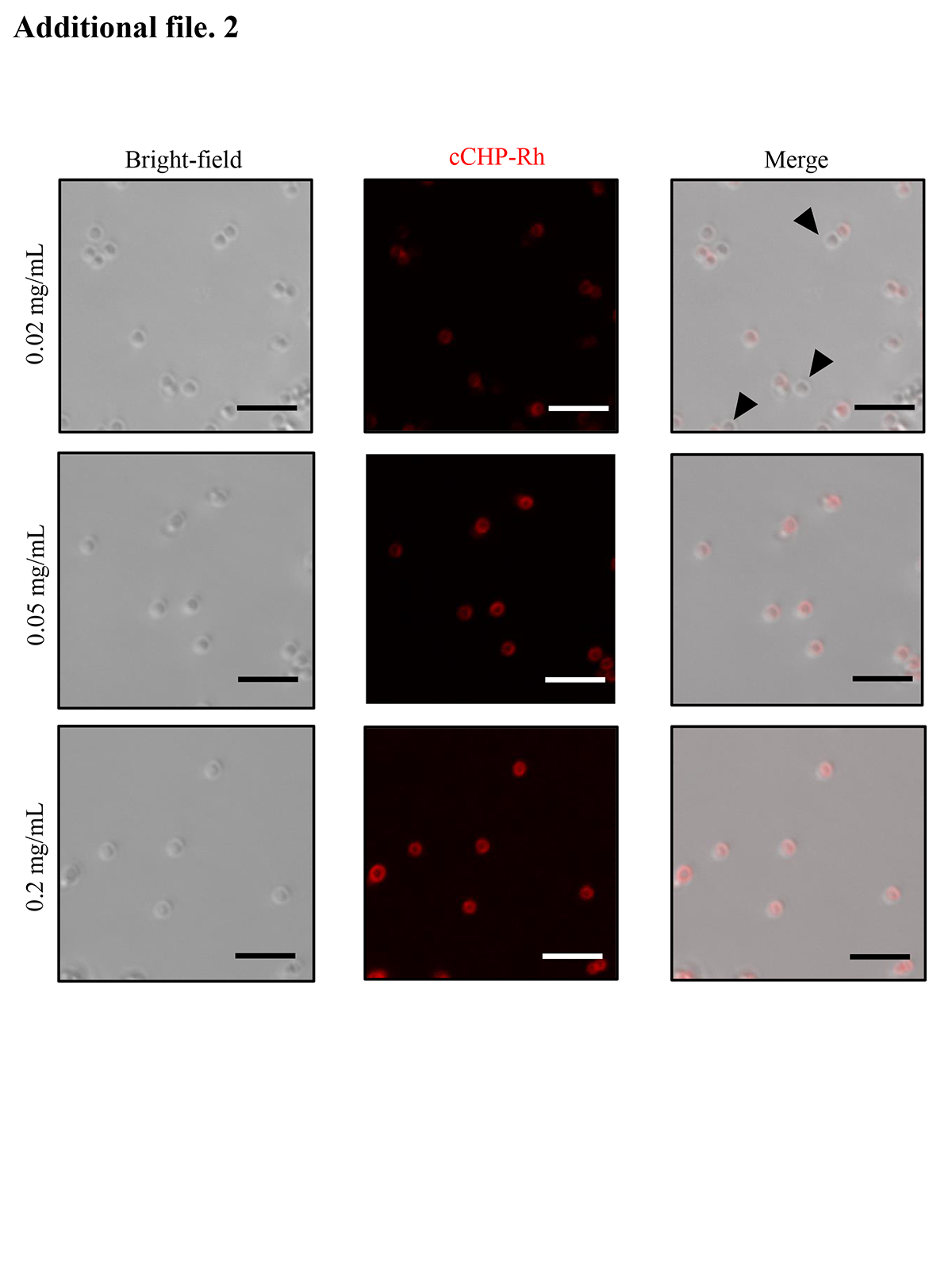

Supplement: Supplementary file 2 — Confocal fluorescence images of inactivated Staphylococcus aureus BM1006 strain incubated with cCHP nanogel. Formalin-killed S. aureus BM1006 samples (FKSA, 5 × 10^9^ cells/mL) were incubated for 30 min at room temperature with 0.02, 0.05, or 0.2 mg/mL cCHP-Rh nanogel, and then observed using a confocal laser microscope. Bright-field, cCHP-Rh, and merge images are shown. Scale bars, 5 μm. Arrows indicate FKSA not complexed by the cCHP-Rh nanogel. (TIF 428 kb) [file 12917_2019_2025_MOESM2_ESM.tif]

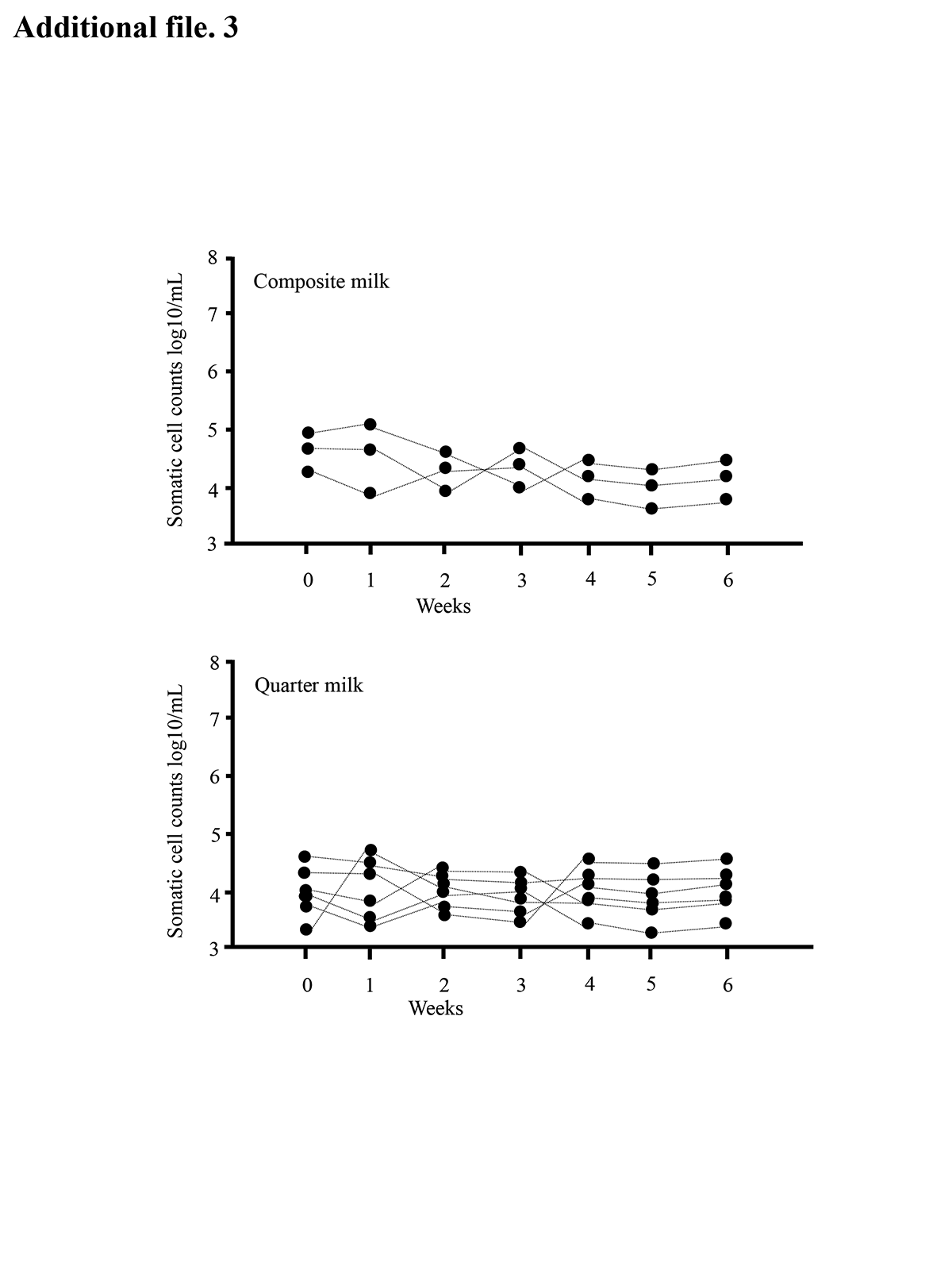

Supplement: Supplementary file 3 — Somatic cell count in milk following nasal immunization with FKSA. After nasal immunization with FKSA or no immunization, somatic cell count (SCC) in composite and quarter milk was analysed. Each data point represents the SCC for composite milk sample (three cows, n = 3) and quarter milk samples (three cows, 12 quarters, n = 12); the bar represents the mean. Black circles correspond to nasal immunization samples. (TIF 82 kb) [file 12917_2019_2025_MOESM3_ESM.tif]
